# What is the optimal strategy in the management of patients with preterm premature rupture of membranes before 32 weeks of gestation?

**DOI:** 10.4274/tjod.48753

**Published:** 2016-03-10

**Authors:** Bilge Çetinkaya Demir, Kiper Aslan, Mehmet Aral Atalay

**Affiliations:** 1 Uludağ University Faculty of Medicine, Department of Obstetrics and Gynecology, Bursa, Turkey

**Keywords:** Chorioamnionitis, prematurity, latency period, expectant management

## Abstract

**Objective:**

Our aim was to compare the outcomes of expectant management of pregnancy or immediate delivery in patients with preterm premature rupture of membranes (PPROM) between 24+^0^ and 32+^0^ weeks of pregnancy.

**Materials and Methods:**

This is a retrospective cohort study conducted at a tertiary medical center. Patients who were diagnosed as having PPROM between 24+^0^ and 32+^0^ weeks of gestation were selected from an electronic database. Thirty-one patients with expectant management and 22 patients with spontaneous immediate delivery were analyzed. Birth weight, Apgar score, duration of stay in the neonatal intensive care unit (NICU), composite adverse outcomes, and mortality rates of groups were compared. Binary logistic regression analysis with backward stepwise elimination was used to determine confounding factors for antenatal complications and neonatal composite adverse outcomes.

**Results:**

Gestational age at admission was smaller in the expectant management group. The median latency period was 6 days (range, 2-58 days). Although gestational age at delivery was similar, birth weights were smaller in expectant management group compared with the immediate delivery group (p=0.264 and p<0.05, respectively). Apgar scores, duration in the NICU, composite adverse outcomes, and neonatal mortality rates were similar in each group. Antenatal complication in the expectant management group was higher (p<0.05). Gestational age at delivery and serum C-reactive protein levels were two confounding factors for antenatal complication and gestational age at delivery was the only factor affecting composite adverse outcome.

**Conclusion:**

Expectant management in patients with PPROM at 24 to 32 gestational weeks might be considered as a good alternative.

## INTRODUCTION

Rupture of fetal membranes before the 37^th^ week of gestation is defined as preterm premature rupture of membranes (PPROM) and is related with a high-risk perinatal period. PPROM occurs in approximately 3% of all pregnancies^(1)^. The most important factor that determines the risk of perinatal morbidity and mortality is gestational age at delivery^(2)^. Perinatal mortality is higher in cases of PPROM before the 32^nd^ gestational week^(3)^. Thus, trying to postpone the delivery is the main concern in these cases by expectant management. There are various studies regarding this issue. Use of antibiotics and corticosteroids and periodic assessment of fetal wellbeing following hospitalization are suggested management plans for these cases^(3)^. Nevertheless, PPROM could increase maternal and fetal risks such as intrauterine infections, maternal sepsis, neonatal sepsis, and fetal demise^(3)^. Therefore, there is a delicate balance between the benefits and risks of expectant management. With expectant management there is no consensus on the optimal delivery time, latency period, and frequency of assessment. The duration of the expectant period ranges from 7 to 10 days in different studies^(4)^. Randomized trials with regard to expectant management generally compare pregnancies between 34 and 37 weeks^(5)^. In this study, we aimed to compare expectant management with immediate delivery of fetuses and to assess which strategy was optimal in management of patients with PPROM between the 24^th^ and 32^nd^ weeks of gestation because the data in the literature concerning expectant management during this period is not strong. Additionally, we tried to determine factors that interfere with maternal and neonatal morbidity and mortality in this period of pregnancy.

## MATERIALS AND METHODS

This study is a retrospective cohort study conducted in a tertiary medical center. The delivery data between January 2009 and January 2015 was retrieved from the hospital’s electronic database and patients who were admitted with a symptom of amniotic fluid gush between the 24^th^ and 32^nd^ weeks of gestation were enrolled.

PPROM was defined as rupture of membranes before the 37^th^ week of gestation; the diagnosis was established in the patient history through speculum examination or placental alpha microglobulin-1 protein (AmniSure, Qiagen Sciences, Germantown, MD, US) testing of vaginal discharge. Gestational age was calculated using the last menstrual period or through first trimester fetal crown-rump length measurement. Patients with placental abruption, chorioamnionitis diagnosed at admission, pre-eclampsia, multiple pregnancies, fetal intrauterine growth retardation, and fetal congenital and chromosomal abnormalities were excluded from study. All patients who were diagnosed as having PPROM between gestational weeks 24 and 32 were hospitalized. Patients were treated with intravenous antibiotics (ampicillin 1 gr, i.v., q.i.d. for 7 days) and antenatal corticosteroid (betamethasone, 12 mg i.m., twice dose at a 24-hours apart). Clinical parameters and laboratory parameters of chorioamnionitis including white blood cell (WBC) counts and C-reactive protein (CRP) testing were evaluated. Tocolysis was administered for only 48 hours during the corticosteroid administration period. Calcium channel blockers and beta-adrenergic receptor agonists are the two preferred tocolytics in our institution. If the patient was in true labor and delivered within 24 hours of admission despite tocolysis, they were included in the immediate delivery group. Patients who did not deliver spontaneously within 24 hours of admission were included in the expectant management group. For the latter group after initial evaluation, maternal fever and heart rate were measured every 6 hours and WBC counts and CRP were monitored every other day. Patients in the expectant management group were not treated with tocolysis beyond 48 hours. Fetal wellbeing was assessed on a daily basis using electronic fetal monitoring and ultrasonography.

Latency period was defined as the time elapsed between onset of PPROM to delivery. The latency period was then divided into 2 subgroups for assessment of clinical outcomes as follows; 1) ≤7 days of latency and 2) >7 days of latency. Delivery eventuated spontaneously or was indicated for non-reassuring fetal status, abdominal pain, placental abruption, and clinical signs or when laboratory findings of chorioamnionitis were observed. Chorioamnionitis was diagnosed on a clinical basis with maternal fever (>38 °C), leukocytosis, uterine tenderness, fetal tachycardia or foul-smelling amniotic fluid with no other source of infection. The attending obstetrician determined the type of delivery depending on patient’s obstetrics history and fetal status.

Antenatal complications that were evaluated in our study included chorioamnionitis, abruption of placenta, and cesarean section due to acute fetal distress or umbilical cord prolapse.

Following delivery, neonates were assessed by a neonatologist and hospitalized for clinical evaluation. Neonatal data including birth weight, Apgar scores, duration of stay in the neonatal intensive care unit (NICU), and composite adverse outcome and mortality were reviewed. Composite adverse outcome comprised conditions such as respiratory distress syndrome (the presence of two or more of the following criteria: evidence of respiratory compromise, a persistent oxygen requirement for more than 24 h, administration of an exogenous surfactant and radiographic evidence of hyaline membrane disease), intraventricular hemorrhage, retinopathy of prematurity, necrotizing enterocolitis and sepsis (proven by bacterial culture of clinical highly suspected sepsis).

Data analysis was performed using SPSS software version 22 (SPSS Inc, Chicago, IL). Continuous data are given in mean with standard deviation or median with minimum and maximum values depending on the distribution characteristics. Categorical data are presented as the number of patients or percentage as appropriate. Student’s t-test and Chi-square tests were used for comparisons between the groups. Binary logistic regression analysis with backward stepwise elimination was used to determine confounding factors for antenatal complications and neonatal composite adverse outcomes. A p value of <0.05 was considered significant.

## RESULTS

**Figure 1:**
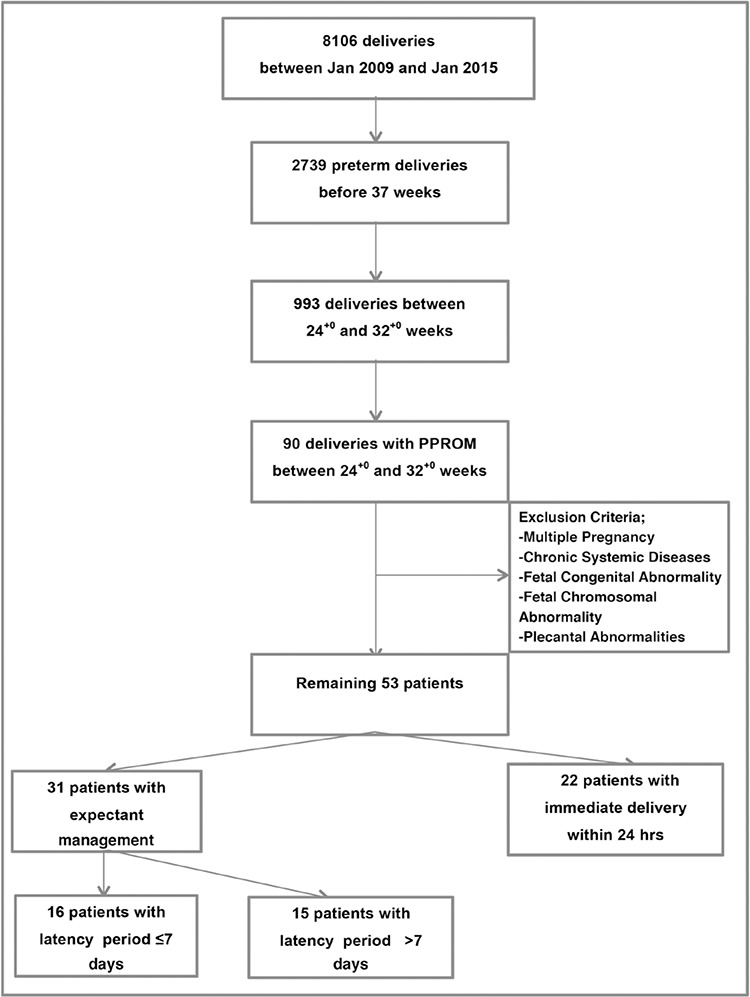
Patient selection flow diagram

During the study period, among the 993 preterm deliveries performed in our institution, 90 were diagnosed as PPROM between the 24^th^ and 32^nd^ gestational weeks. Fifty-three patients remained in the final analysis after application of exclusion criteria (Figure 1).

Among the 53 patients, 22 delivered within 24 hours (immediate delivery group) and 31 patients were managed expectantly. The maternal characteristics were similar in both groups (Table 1). Median maternal age of the immediate delivery group and expectant management group was 30 years (range, 18-37 years) and 30 years (range, 21-42 years), respectively. Gestational age at admission was 218 days (range, 182-224 days) for the immediate delivery group and 189 days (range, 168-224 days) for the expectant management group (p<0.05). The mean WBC and C-reactive protein measurements were similar in each group at admission. For the expectant management group, the median interval time was 6 days (range, 2-58 days). Gestational age at delivery was similar; 218 days (range, 182-224 days) for the immediate delivery group and 207 days (range, 176-267 days) for the expectant management group. There was no fetal demise.

There were more antenatal complications in the expectant management group compared with the immediate delivery group (48% vs 18%, respectively) (p<0.05) (Table 1). However, patients with a latency period >7 days did not have significantly increased maternal complication rates compared with patients with a latency period of ≤7 days (40% vs 56%, respectively) (p=0.479) (Table 2). No patients delivered due chorioamnionitis with <7 days of latency. Three out of 6 patients with a latency period >7 days delivered due to chorioamnionitis. Two patients were delivered due to non-reassuring fetal status.

Antenatal complication was a dependent variable, and gestational age at admission, gestational age at delivery, interval day, WBCs at delivery and CRP at delivery were independent variables in the binary logistic regression analysis. Gestational age at delivery and CRP levels at delivery were found to be 2 confounding factors for increased antenatal complications (Predicted logit of antenatal complication=8.637+(-0.046) x gestational age at delivery + 0.162 x (CRP at delivery) (Omnibus test of model: p<0.001; Nagelkerke R Square=0.251; Hosmer-Lemeshow goodness of fit test p=0.487).

Neonatal birth weight was larger in the immediate delivery group compared with the expectant management group (1613±452 g vs. 1274±429 g, respectively). First and 5^th^ minute Apgar scores were similar for both groups (Table 1). The median duration of stay in the NICU was statistically similar in both groups; 25 days (range, 1-90 days) for the immediate delivery group and 30 days (range, 1-130 days) for expectant management group (Table 3). The percentage of ventilation requirement in neonates was similar in both groups (47% vs. 72% respectively) (p=0.08). Composite adverse outcomes for both groups were also similar (p=0.498). Although neonatal mortality was more common in the expectant management group compared with the immediate delivery group (35% vs. 14%, respectively), it did not reach statistical significance (Table 3). Neonatal outcomes were also similar in the subgroups of the expectant management group (Table 4). Two out of 3 neonates born to mothers with chorioamnionitis expired; only one could be discharged from the NICU.

When binary logistic regression analysis with backward stepwise elimination was conducted, gestational age at delivery was found as the only factor related with neonatal composite adverse outcome. The other variables, which were gestational age at admission, latency period, WBCs and CRP at delivery, betamethasone use and birth-weight, were not found as factors related to fetal and maternal morbidity. (Predicted logit of composite adverse outcome=37.266+(-0.162) x gestational age at delivery (Omnibus test of model: p<0.001; Nagelkerke R Square=0.700; Hosmer-Lemeshow goodness of fit test p=0.553).

## DISCUSSION

Management of PPROM with regard to gestational age is a challenging issue. Immediate delivery is the preferred method with acceptable outcomes because fetal lung maturity is almost achieved after 34 weeks of gestation. However, if the gestational age is smaller than 32 weeks, the management becomes more complex and difficult. While dealing with these PPROM cases, one should weigh the risks of immediate delivery and expectant management. If immediate delivery is the choice, neonatal complications and mortality related with prematurity of newborn are the main disadvantages. If expectant management is the strategy of choice, the risks of chorioamnionitis and neonatal sepsis are the two arising morbidities. In order to overcome these risks and to have the best neonatal outcomes, we need to increase our knowledge about the optimal expectancy period.

Our study demonstrated that gestational age in PPROM is inversely associated with duration of latency. Patients with a shorter gestational age had a longer latency period until delivery. The antenatal complication rate was found higher with prolongation of pregnancy. However, expectant management in PPROM between 24 and 32 weeks was not associated with increased neonatal morbidity and mortality compared with the immediate delivery group. Gestational age at delivery was the only confounding factor for composite adverse outcomes.

Similar to our study, Aziz et al.^(6)^ demonstrated that gestational age at the time of PPROM was inversely associated with duration of latency. Nevertheless, there was no consensus on length of latency to optimize the neonatal morbidity. Peaceman et al.(^7)^ assessed the optimal length of latency in patients with PPROM before 32 weeks of gestation in a multicenter randomized study. The authors found that with expectant management, the optimal latency period was approximately 9 days before 28 weeks of gestation, but was significantly shorter in patients with 29-30 weeks of gestation^(7)^. In another study for all gestational age groups between 24-34 weeks, the best outcomes were in patients with PPROM of 1 to 7 days, and survival was lowest in infants where PPROM was >28 days^(8)^.

Manuck et al.^(9)^ found that in patients with PPROM, infection but not latency was associated with major perinatal morbidity. In another study, it was found that composite neonatal morbidity in neonates born with a latency period >7 days was higher compared with the composite morbidity of neonates born at the same gestational age without PPROM^(10)^. The reason for the higher rate of adverse neonatal outcomes in cases of uncomplicated PPROM is unclear. One possible explanation may be the presence of subclinical chorioamnionitis, which has been shown to complicate up to 25-40% of cases of PPROM^(11)^. However, other studies have shown the majority of infants with prolonged latency periods following PPROM did not have increased morbidity and mortality risk in the NICU compared with similar gestational age infants born to mothers with a shorter latency period of PPROM, similar to our results^(6,8)^. Gestational age was the only confounding factor for composite adverse neonatal outcomes. Thus, we argue that expectant management might be considered as a good alternative in the management of patients with PPROM <32 weeks.

Although there was no statistical difference in gestational age at delivery, the mean birthweight was lower in the expectant management group compared with the immediate delivery group. This may be attributed to the presence of oligohydramnios and inflammatory process in the prolonged latency period. The literature about birthweight is conflicting. Some studies showed that oligohydramnios at admission was associated with adverse neonatal outcomes^(12,13)^. In contrast, others reported that oligohydramnios was not related with fetal growth restriction^(14)^. Subclinical chorioamnionitis has also been shown to be responsible for fetal growth restriction^(15)^. In our study, WBC was not statistically higher in the expectant management group at delivery. CRP level at birth was one of the confounding factors for maternal complications but not in neonatal composite adverse outcomes. It is one of the clinical findings in subclinical chorioamnionitis, which may also explain the presence of lower fetal weight in these patients. Additionally, the shorter gestational age at admission in the expectant management group may be another reason for the lower birthweights compared with the immediate delivery group.

This small-sample-sized retrospective study showed that expectant management may be considered before 32 weeks of gestation in appropriate patients. Optimizing maternal-fetal management with a conservative treatment toward prolonging pregnancy for as long as possible seems pragmatic because antenatal and neonatal complications are all effected by gestational age. We conclude that further randomized and larger trials are required to establish the optimum length of the latency period in this particular condition.

## Figures and Tables

**Table 1:**
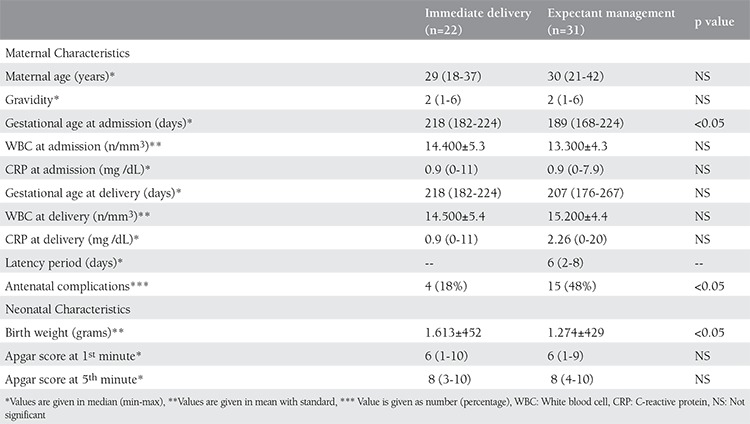
Maternal and neonatal characteristics of the patients

**Table 2:**
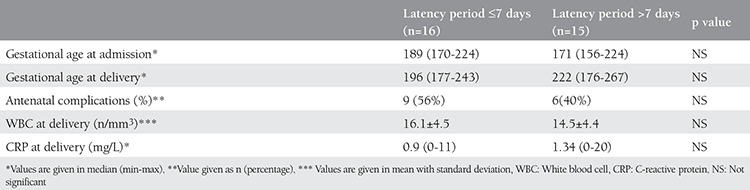
Comparisons between subgroups of patients with expectant management

**Table 3:**
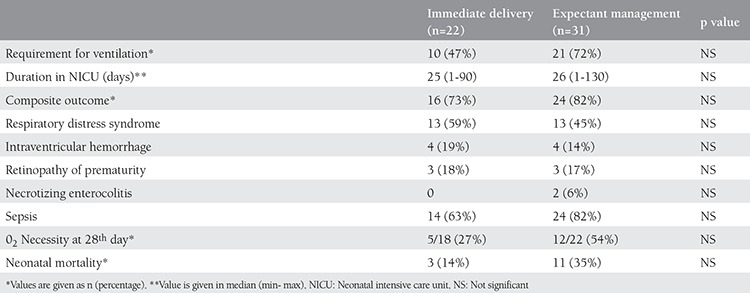
Comparisons between neonatal outcomes

**Table 4:**
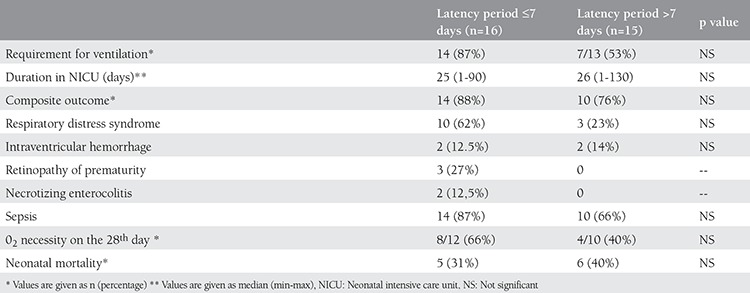
Comparisons of neonatal outcomes between subgroups of patients with expectant management
